# Hydromorphone reduced the incidence of emergence agitation after adenotonsillectomy in children with obstructive sleep apnea: A randomized, double-blind study

**DOI:** 10.1515/med-2024-1129

**Published:** 2025-02-07

**Authors:** Qiyuan Huang, Yang Chen, Xiaohui Sun, Yongwei Su, Ruihao Zhou, Guo Chen, Tao Zhu

**Affiliations:** Department of Anesthesiology, West China Hospital, Sichuan University, Chengdu, 610041, Sichuan, China; Department of Anesthesiology and Operation Center, West China Hospital, Sichuan University/West China School of Nursing, Sichuan University, Chengdu, Sichuan, China

**Keywords:** emergence agitation, adenotonsillectomy surgery, hydromorphone, fentanyl, children

## Abstract

**Purpose:**

Emergence agitation (EA) after (adeno)tonsillectomy (AT) surgery impairs recovery in children. Adequate analgesia plays a crucial role in reducing EA incidence. This study investigated whether hydromorphone infusion (30 μg/kg) during anesthesia induction could reduce EA following AT surgery for obstructive sleep apnea in children.

**Patients and methods:**

A total of 186 ASA I–III children aged 3–7 years undergoing AT surgery were enrolled in a blinded randomized trial comparing hydromorphone (30 μg/kg) to fentanyl (4 μg/kg). The primary outcome was EA incidence within 30 min post-extubation. Secondary outcomes included pediatric anesthesia emergence delirium (PAED), face, legs, activity, crying, consolability (FLACC), Ramsay sedation scores, extubation time, rescue analgesia incidence, and adverse events.

**Results:**

The incidence of EA was significantly lower in the hydromorphone group [48.4% (45/93) vs 64.5% (60/93); absolute difference: 16.1%; 95% CI: 18.9–29.5%; *P* = 0.027]. Hydromorphone improved PAED, FLACC, and Ramsay scores and reduced moderate-to-severe pain and rescue analgesia. No postoperative complications occurred in either group.

**Conclusion:**

Hydromorphone at 30 μg/kg effectively reduces the incidence of EA within 30 min post-extubation in children after AT surgery compared to fentanyl. It shows superior analgesia and has a low incidence of adverse effects.

## 1 Introduction

Pediatric obstructive sleep apnea (OSA) is a high socioeconomic and humanistic burden for both family and public health [1,2]. (Adeno)tonsillectomy (AT) used to be performed based on the “focal infection” theory that the tonsils were the portal of entry for these infections. In recent decades, as the understanding of the negative effects of upper airway obstruction comprehended, AT surgery has emerged as the first-line minimal invasive treatment for OSA.

Pain and discomfort following surgery are known triggers for emergence agitation (EA). AT surgery is an independent risk factor of postoperative EA in pediatrics, attributed to moderate to severe postoperative pain and intense discomfort at the surgical site [3]. In younger pediatric patients, particularly those aged 3–5 years, the incidence of EA following AT surgery varies between 25.5 and 63.3%, exceeding the average incidence of post-general anesthesia delirium in this popularity [4–8]. Although transient (typically within the first 10–30 min after surgery), EA significantly hinders enhanced recovery after surgery. It is associated with several complications, including an increased risk of postoperative bleeding [9,10], prolonged recovery times, and early postoperative negative behaviors such as sleep disturbances and separation anxiety [11]. Therefore, optimizing analgesic protocols to treat EA in children undergoing AT surgery is crucial, but still challenging.

Earlier survey study suggested diverse approaches to prevent and treat EA in children using opioid analgesics [12], α_2_-adrenergic receptor agonists [13], or propofol [14,15]. Nevertheless, consideration must be given to unperceived postoperative airway obstruction and severe respiratory depression in children with OSA, especially when using opioid analgesics [16]. Fentanyl is a commonly administrated opioid analgesic during induction of general anesthesia in children, and it significantly decreases the incidence of EA in children undergoing general anesthesia [17,18]. Hydromorphone, as an opioid analgesic with a potent and relatively long-lasting effect compared to fentanyl, can potentially reduce postoperative pain more effectively, which in turn may decrease the incidence of EA by mitigating one of its primary causes [19–21]. The administration of 5–30 μg/kg hydromorphone (intravenous injection) during induction of general anesthesia showed good acute pain management and low incidence of adverse respiratory events in children [22,23]. Other studies have shown that an infusion of hydromorphone by nurse/caregiver-controlled analgesia provides satisfactory analgesia and demonstrates a high safety profile in diverse postoperative settings [24]. Literature on using hydromorphone during induction of general anesthesia is still sorely lacking, especially among children undergoing AT surgery.

To test the hypothesis that administrating hydromorphone as part of the anesthesia induction regimen could better control postoperative EA in children who underwent AT surgery, we conducted a prospective controlled trial to measure the difference in incidence of EA between groups assigned to hydromorphone infusion (30 μg/kg) and fentanyl infusion (4 μg/kg).

## 2 Methods

### 2.1 Study design

This prospective, randomized, double-blind clinical trial was conducted at the West China Hospital, a tertiary teaching hospital, from July 15, 2020 to July 15, 2022. Patient enrollment began on November 12, 2020 to December 31, 2021.

### 2.2 Population

The study population comprised children scheduled for AT surgery. Children of both sexes with an American Society of Anesthesiologists (ASA) physical status of I, II and III, ages 3–7 years, were included. The exclusion criteria were patients who had contraindications to general anesthesia, or allergy to any medication of regimens of both groups, or who has psychomotor disorder/retardation that precluded adequate communication, or whose parents had conditions that precluded adequate communication during follow-up visits.

### 2.3 Randomization and blinding

Patients and all study personnel except the investigative pharmacist at each site were blinded to the treatment assignment. Eligible patients were randomized 1:1 to receive hydromorphone to obtain more comprehensive safety data during anesthesia induction use. Fentanyl was selected as the comparator medication because it was commonly used for induction of anesthesia and perioperative analgesia in children in many countries [22,25,26], including China [12]. All patients were randomized using a computer-generated schedule. Detailed information regarding baseline demographics, BMI, and ASA physical status was obtained at the time of enrollment after consent was signed.

One nurse prepared all the study drugs and syringe pump systems for micro-infusion, and this nurse was not involved in the surgical procedure or postoperative care. Hydromorphone and fentanyl were diluted with the same 20 mL of saline in each group, so it was not possible to distinguish the drugs by the appearance of the syringe.

The allocation was concealed by providing sequentially coded and numbered syringe packets of study medications, labeled with serial numbers and no identifiers for the medication. The study packets (containing prepared study medication syringes) were delivered to the attending anesthesiologist by a research assistant. The research assistant was not aware of the allocation sequence and was also blinded to the study medications. The research assistant would attach the medication sequence number on the patient case report form records and write down the patient’s hospital ID on the medication record log. Since the medication syringes contained clear solutions of study medications in equivalent volume, the anesthesiologists, patients, PACU nurses, and research assistants were effectively blinded.

The patient’s adverse effects were monitored by the nurse in PACU and ward. If the child had any adverse effects, a blinded anesthesiologist was immediately notified for appropriate management.

### 2.4 Anesthetic regimen and study drug administration

Premedication was not administered. Under standard monitoring (electrocardiogram, pulse oximeter oxygen saturation, non-invasive blood pressure, end-tidal carbon dioxide, and the bispectral index [BIS]), we induced anesthesia with 0.05 mg/kg midazolam (maximum 1 mg) and 2–3 mg/kg propofol, accompanied by dexamethasone 0.25 mg/kg. Analgesics during induction were administrated as follows: patients in fentanyl group received 20 min of micro-infusion of fentanyl (4 μg/kg fentanyl in total diluted in 20 mL 0.9% sodium chloride); whereas patients in hydromorphone group received 30 μg/kg hydromorphone [23] in the same way.

Tracheal intubation was facilitated with 0.1–0.15 mg/kg cis-atracurium, then mechanically controlled ventilation was used to maintain end-tidal carbon dioxide at 35 ± 5 mmHg during the surgery. Anesthesia was maintained with 1.0–4.0 vol% end-tidal sevoflurane in an air-oxygen mixture (fraction of inspired oxygen = 0.5) and micro-infusion of remifentanil at 0.15–0.2 μg/kg/min. The speed of remifentanil infusion and concentration of end-tidal sevoflurane was adjusted according to the clinical parameters (blood pressure or heart rate within 20% of the baseline and BIS of 40–60). Normal saline solution was administered for fluid management (according to 4/2/1 rule) to all the patients. The same otolaryngologist performed all surgical procedures to maintain a uniform application of the surgical stimulus. Sevoflurane and remifentanil were discontinued at the end of the operation. We antagonized neuromuscular blockade with intravenous neostigmine 0.05 mg/kg and atropine 0.01 mg/kg. During the operation, normal blood volume was maintained via 0.9% sodium chloride–2.5% glucose mixed solution. The intraoperative warming was conducted via a warm blanket set at 42°C.

Ten  minutes before the end of the operation, we administrated ondansetron 0.15 mg/kg to prevent nausea or vomiting. Before being transferred to the PACU, children received 1–2 mg/mL propofol to prevent accident endotracheal extubation during transfer. The wake-extubation technique was performed in the PACU. After the child awakened, popsicles were given at the child’s request to help control postoperative pharyngeal pain. Before transferring to the ward, all patients were evaluated with the modified Aldrete score and reached at least 9 points.

### 2.5 Outcomes

The primary outcome was the incidence of EA within 30 min after extubation evaluated using the Pediatric Anesthesia Emergence Delirium (PAED) scale [27] (Table 1). PAED scale ≥12 was defined as EA. If the children with EA denied crying due to pain and reassuring words was inconsolable, a single dose of propofol (1 mg/kg) was administered intravenously to treat EA. After 5 min of propofol administration, children with PAED scales still above 12 points received a single dose of propofol again.

**Table 1:** PAED

Descriptions	Not at all	Just a little	Quite a bit	Very much	Extremely
The child makes eye contact with the caregiver	4	3	2	1	0
The child shows purposeful actions	4	3	2	1	0
The child is aware of his or her surroundings	4	3	2	1	0
The child is restless	0	1	2	3	4
The child is inconsolable	0	1	2	3	4

The secondary outcomes were as follows:

(a) EA: incidence of EA and PAED scale at 0, 10, 20, 30 min after extubation, before leaving PACU, and 2, 4, 6 h after operation.
(b) Postoperative pain: the face, legs, activity, crying, consolability (FLACC) scale at 0, 10, 20, 30 min after extubation, before leaving PACU, and 2, 4, 6 h after operation; mild pain: FLACC = 1–3, moderate pain: FLACC = 4–6, severe pain: FLACC = 7–10 [28].
(c) Sedation: Ramsay scales at 0, 10, 20, 30 min after extubation, before leaving PACU, and 2, 4, 6 h after operation.
(d) Extubation time: the time between discontinuation of anesthesia and that taken to meet extubation criteria, i.e., regular respiration, grimaces, and/or purposeful movements [8].
(e) Duration of PACU stay: the time from the end of surgery to discharge from the PACU.
(f) Incidence of rescue analgesia or sedation in PACU: when the FLACC score >3, children in the fentanyl group received intravenous injections of 0.5 μg/kg fentanyl. In the hydromorphone group, hydromorphone was intravenously administrated at 5 μg/kg.
(g) Side effects: incidence of postoperative nausea and vomiting, respiratory depression, oversedation, desaturation, constipation, urinary retention, or severe itching within 24 h after surgery.

### 2.6 Sample size and statistical analyses

The primary endpoint of this study was EA incidence within 30 min after extubation. The incidence of EA varies between 25.5 and 63.3% in children following AT surgery [4–8]. At our institution, the incidence of EA after fentanyl-sevoflurane anesthesia for pediatric AT surgery was 55%. At a design inspection efficiency (1−*β*) = 0.8, inspection level *α* = 0.05 (bilateral), if we expected a 40% reduction in the incidence of EA with hydromorphone treatment (33% of the children in the hydromorphone group may have EA), 84 patients in each group were required. Assuming a 10% dropout rate, the final sample size was set at 186 patients (93 patients per group).

We analyzed outcomes according to a modified intention-to-treat principle in the full analysis set. Children who received the allocated intervention and had at least one data point after treatment were included in the full analysis set. Distributions of data were first examined for normality. Dichotomous outcomes were reported as numbers and percentages for each group, and odds ratio to compare between groups. 95% confidence intervals were reported for precision. For continuous variables, Shapiro–Wilk test was used for the normal distribution test and Levene’s test was used to test the homoscedasticity assumption. Data that retained both assumptions were analyzed by the independent sample *t*-tests. Otherwise, Mann–Whitney *U* tests were used to compare medians. Categorical variables were analyzed with chi-square tests with continuity correction or Fisher exact tests. For all analyses, a two-sided test with *α* = 0.05 was designated as significant. Statistical analyses were performed using SPSS (IBM SPSS Statistics, version 25.0).


**Informed consent:** Informed consent was obtained from the parents/legal guardians of the study participants prior to study commencement.
**Ethical approval:** This study has been approved by the Ethics Committee on Biomedical Research, West China Hospital of Sichuan University, China (ethical approval number 2020-459). The study complied with guidelines of the Consolidate Standard of Reporting Trials (CONSORT) checklist and was retrospectively recorded at the Chinese Clinical Trials Register (www.chictr.org.cn); registration number: ChiCTR2000039914.

## 3 Results

### 3.1 Demographic, surgery, and anesthesia data

**Figure 1:**
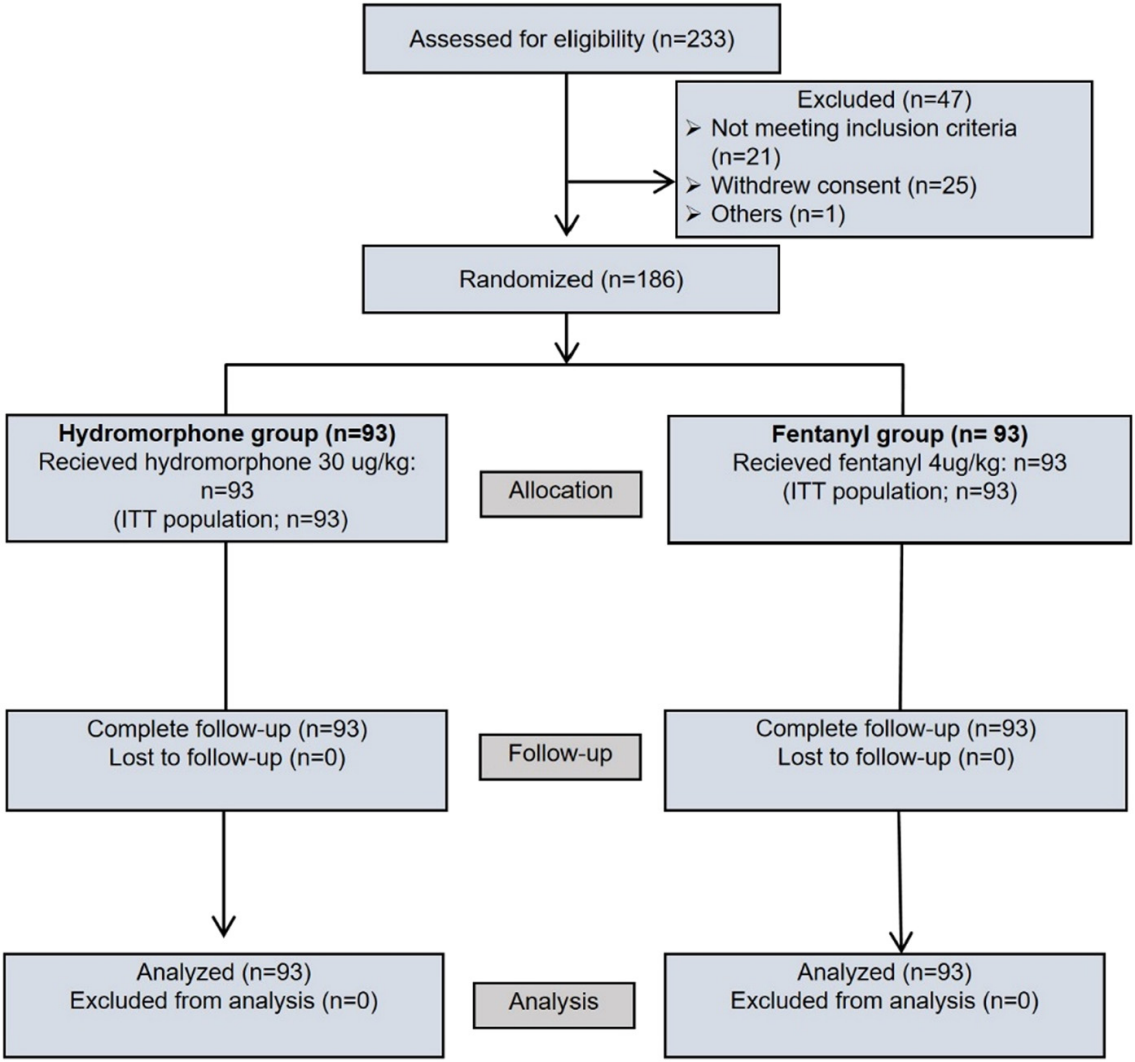
Consolidated standards of reporting trials flow diagram.

A total of 233 patients were screened for eligibility. 47 patients were excluded since they did not meet the inclusion criteria or refused to participate. 93 patients in the hydromorphone group and 93 patients in the fentanyl group received the intervention and were included in the statistical analysis (Figure 1). There were no significant differences in demographics, ASA status, modified Yale preoperative anxiety scale (m-YPAS) scores, or duration of anesthesia or operation (*P* > 0.05) (Table 2). However, the duration of PACU stay (60.4 ± 14.3 vs 55.1 ± 11.5, *P <* 0.01) and extubation time (37.7 ± 12.5 vs 30.5 ± 10.9, *P <* 0.0001) in hydromorphone group was significantly longer than that in the fentanyl group.

**Table 2:** Baseline participant characteristics

Characteristic	Hydromorphone group (*n* = 93)	Fentanyl group (*n* = 93)	*P-*value^a,b^
Age (years)	4.9 ± 1.3	5.3 ± 1.3	0.057
Sex			0.100
Male	50 (53.8)	61 (65.6)	
Female	43 (46.2)	32 (34.4)	
Weight (kg)	19.4 ± 4.4	20.3 ± 4.9	0.190
Height (cm)	111.6 ± 10.1	114.4 ± 10.3	0.069
BMI (kg/m^2^)	15.4 ± 1.8	15.3 ± 2.0	0.766
ASA status			0.989
I	5 (5.4)	4 (4.3)	
II	87 (93.5)	89 (95.7)	
III	1 (1.1)	0	
m-YPAS	35.9 ± 5.2	36.2 ± 6.7	0.791
Duration of anesthesia (min)	66. 0 ± 15.4	69.1 ± 20.5	0.249
Duration of surgery (min)	34.3 ± 13.4	38.8 ± 17.9	0.053
Dosage of remifentanil (μg)	172.8 ± 83.0	190.7 ± 104.2	0.196
Extubation time (min)	37.7 ± 12.5	30.5 ± 10.9	**<0.0001**
Duration of PACU stay (min)	60.4 ± 14.3	55.1 ± 11.5	**<0.01**

Values are mean ± SD or number (%).

Abbreviations: SD, standard deviation; BMI, body mass index; ASA, American Society of Anesthesiologists; m-YPAS, modified Yale preoperative anxiety scale; PACU, post-anesthesia care unit. ^a^ *P* value compares the hydromorphone group and fentanyl group. ^b^ *t*-test used to compare mean values and chi-squared test used to compare proportions; the Whitney–Mann *U* test was used for the comparison of ordinal variables.

The data are bolded to highlight the values with a *p*-value less than 0.05, indicating statistical significance.

### 3.2 Primary outcome

A cut-off PAED score of ≥12 was defined as EA [27]. The incidence of EA within 30 min after extubation was significantly lower in the hydromorphone group compared to the fentanyl group [48.4% (45/93) vs 64.5% (60/93); absolute difference, 16.1%; 95% confidence interval, 18.9–29.5%; *P* = 0.027].

#### 3.2.1 Components of the primary outcome

The incidence of EA in the hydromorphone group was significantly lower than that in the fentanyl group at 10, 20, and 30 min after extubation and before leaving PACU (Table 3). The trend Chi-square test was used to analyze the trend of the incidence of EA at nine different time points in both groups. The trend *P*-values were lower than 0.001, suggesting a decreasing trend in the incidence of EA after transferring to PACU in both groups (Table 3).

**Table 3:** Comparison of incidence of emergence agitation between two groups at different time points after transferring to PACU

Time points/subgroups	Hydromorphone group, *n* (%)	Fentanyl group, *n* (%)	OR (95% CI)	*P-*value^a,b^
**Emergence agitation within 30 min after extubation**				
All subjects	45/93 (48.4)	60/93 (64.5)	1.94 (1.08–3.49)	**0.027**
3–5 years old	30/58 (51.7)	38/49 (77.6)	3.22 (1.38–7.51)	**0.006**
6–7 years old	15/35 (42.9)	22/44 (50.0)	1.33 (0.55–3.26)	0.527
**Emergence agitation at each time point**				
Eyes open	35 (37.6)	43 (46.2)	1.43 (0.79–2.56)	0.235
0 min at extubation	37 (39.8)	36 (38.7)	0.96 (0.53–1.72)	0.881
10 min at extubation	14 (15.1)	32 (34.4)	2.96 (1.45–6.03)	**0.002**
20 min at extubation	7 (7.5)	30 (32.3)	5.85 (2.42–14.17)	**<0.001**
30 min at extubation	7 (7.5)	25 (26.9)	4.52 (1.84–11.07)	**<0.001**
Exiting PACU	3 (3.2)	20 (21.5)	8.22 (2.35–28.75)	**<0.001**
Postoperative 2 h	1 (1.1)	4 (4.3)	4.13 (0.45–37.71)	0.368
Postoperative 4 h	1 (1.1)	1 (1.1)	1.00 (0.06–16.23)	1.000
Postoperative 6 h	1 (1.1)	0	—	1.000
*P* trend^c^	<0.001	<0.001		

Abbreviations: PACU, post-anesthesia care unit; OR, odd ratio; CI, confidence interval. ^a^ *P-*value compares the hydromorphone group and fentanyl group. ^b^Chi-squared test was used to compare proportions. ^c^Chi-squared trend test was used to calculate *P* trend.

In subgroup post-analysis by age, the difference in the incidence of EA between hydromorphone and fentanyl was only observed in children aged 3–5 years old (51.7% vs 77.6%, *P* = 0.006). However, this difference became insignificant in older children aged 6–7 years old (42.9% vs 50.0%, *P* = 0.527).

### 3.3 Secondary outcomes

#### 3.3.1 EA-related outcomes

**Figure 2:**
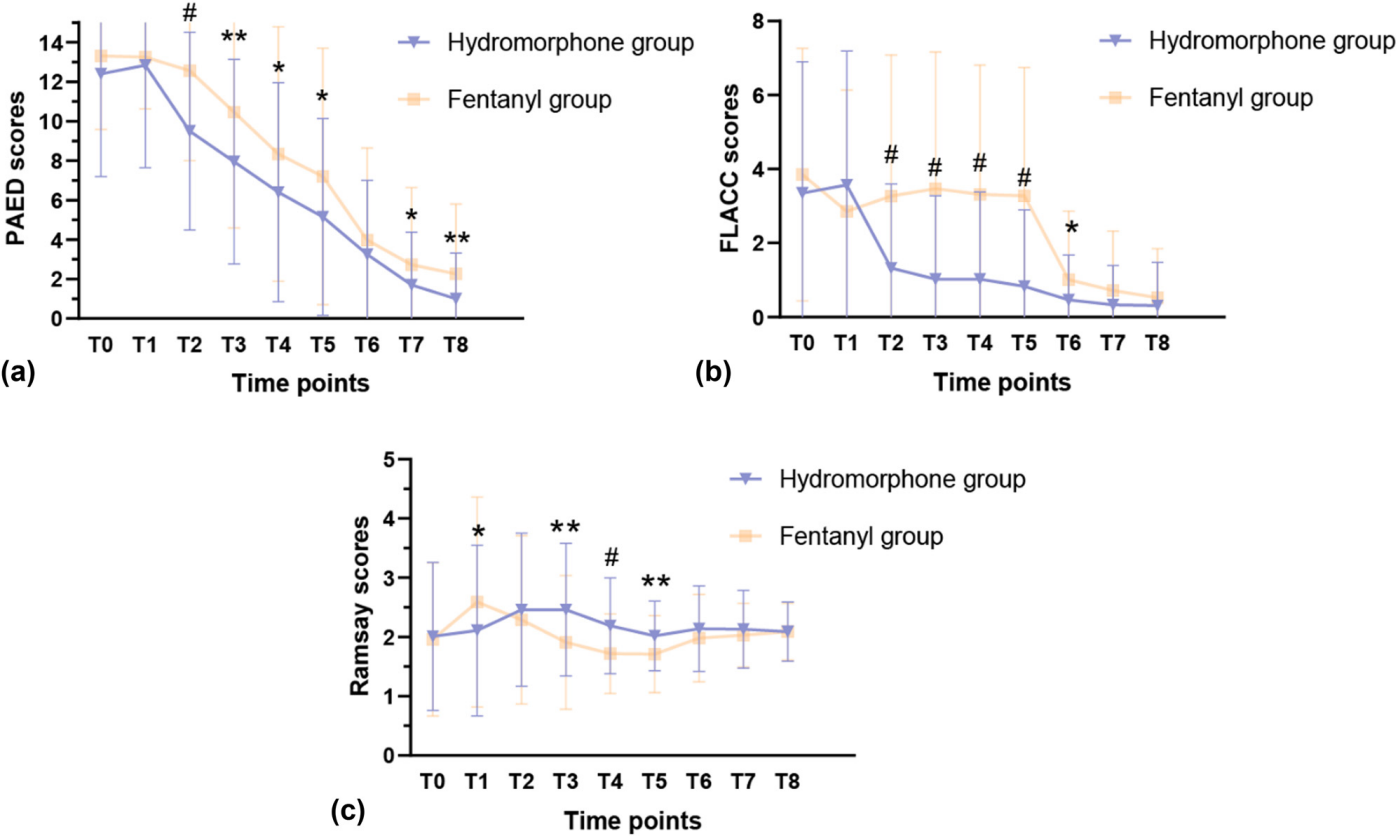
Comparison of (a) PAED, (b) FLACC, and (c) Ramsay scores at T0 (eyes open), T1 (0 min at extubation), T2 (10 min at extubation), T3 (20 min at extubation), T4 (30 min at extubation), T5 (exiting post-anesthesia care unit), T6 (postoperative 2 h), T7 (postoperative 4 h), and T8 (postoperative 6 h) between the hydromorphone group and fentanyl group. ^*^ *P <* 0.05; ^**^ *P <* 0.01; ^#^ *P <* 0.001.

We observed significantly lower mean PAED scores in the hydromorphone group at 10, 20, and 30 min after extubation and before leaving PACU (Figure 2a and Table S1). Although the incidence of EA at postoperative 4 and 6 h were comparable between the two groups, the mean PAED scores were significantly lower in the hydromorphone group.

#### 3.3.2 Pain-related outcomes

Since postoperative pain intensity and sedation play a vital role in the incidence of EA, we evaluated postoperative FLACC scores and Ramsay scale values. The results showed that FLACC scores were lower in the hydromorphone group than in the fentanyl group at 10, 20, and 30 min after extubation and before leaving PACU (Figure 2b and Table S2). The proportion of patients with postoperative moderate-to-severe pain (FLACC scores >3 at any time point) was significantly lower in the hydromorphone group than in the fentanyl group (57.0% vs 77.4%, *χ* ^2^ = 8.806, *P* = 0.003). Ramsay scale scores at 10, 20, and 30 min after extubation and before leaving PACU in the hydromorphone group were lower than those in the fentanyl group (Figure 2c and Table S3).

#### 3.3.3 Rescue analgesia and complications

Only three patients (3.2%) in the hydromorphone group received rescue analgesia, while this number in the fentanyl group reaches 24 (25.8%), and the overall proportion of rescue analgesia was significantly higher in the fentanyl group (3.2% vs 25.8%, *χ* ^2^ = 19.107, *P＜*0.001). Incidences of rescue sedation were comparable between the two groups (5.4% vs 12.9%, *χ* ^2^ = 3.172, *P* = 0.075). Hyperthermia and post-AT hemorrhage in PACU were observed in one child of the fentanyl group. No other adverse effect was observed in all subjects, e.g., postoperative nausea and vomiting, respiratory depression, oversedation, desaturation, constipation, urinary retention, or severe itching.

## 4 Discussion

The most important finding was that the anesthesia induction with hydromorphone infusion was associated with a lower incidence of EA within 30 min after extubation among children undergoing AT surgery.

In children, EA and pain are often present simultaneously. Somaini et al. found that in the early postoperative, 21% of patients had EA, and EA and pain are associated in up to 65% of patients [29]. Therefore, the ideal drug should be administrated preemptively [30], and it should have a reliable postoperative analgesic effect and light respiratory depression effect. This study demonstrated that administration of hydromorphone during induction of anesthesia provided a significantly stronger analgesic effect than fentanyl and reduced the incidence of EA in the early postoperative period. We attributed this strong analgesic effect to the high affinity of hydromorphone for both μ and δ receptors, thus reducing the afferent of injurious stimuli.

In subgroup-analysis by age, our finding aligned well with previous study that showed how incidence of EA varied in different age groups with three times greater risk in pre-school age (less than 6 years) [31]. We believe children aged 3–5 years may benefit more from hydromorphone compared to fentanyl, whereas this advantage may not be as pronounced in older preschool-aged children.

Hydromorphone provides satisfactory analgesia in the current setting. Although the decrease in mean PAED scores was statistically significant in the hydromorphone group, the CI for the difference in mean PAED scores did not fully exceed the clinically important difference (at least 4 points [32]) at any of these prespecified time points. The clinical relevance of this change therefore remains uncertain, and the interpretation of the alleviated PAED scores based on this study should be considered inconclusive.

Among pediatric patients who underwent ambulatory operations the overall opioid use trended downward, while the intravenous administration of fentanyl and hydromorphone increased from 2010 to 2017 [33]. A growing number of studies have investigated the potential of various types of hydromorphone administration. In the present study, children received hydromorphone at a dose of 30 μg/kg by microinjection. Several previous studies have selected 5–200 μg/kg hydromorphone for intravenous administration [22,34,35], continuous infusion of 40–60 μg/h for epidural administration, or 10–15 µg/kg with 2.5–6 µg/kg on-demand for patient-controlled analgesia [20,21]. The inactive metabolite of hydromorphone contributes to its safe administration in a variety of situations. Since the incidence of rescue analgesia is low and no opioid-associated complications occurred in this study, we consider applying hydromorphone by micro-infusion at a dose of 30 μg/kg may be adequate and safe in this setting.

Here, hydromorphone compared to fentanyl prolong the mean extubation time by 7 min and the duration of PACU stay by 5 min, though it did not increase the incidence of postoperative complications. We attribute this result to the longer and stronger analgesic effect of hydromorphone compared to fentanyl. According to previous research, “Extubation time” was defined as the time between discontinuation of anesthesia and that taken to meet extubation criteria, i.e., regular respiration, grimaces, and/or purposeful movements [22]. Therefore, in the fentanyl group, inadequate analgesia could relatively shorten the time to emergence and extubation, causing higher occurrences of EA and moderate-to-severe pain.

One notable benefit of hydromorphone is its potency; it is 5–7 times more potent than morphine [19]. This increased potency allows for smaller dosages while still achieving effective pain control, which may help reduce the risk of opioid-related side effects. In contrast, when compared to non-opioid agents, such as NSAIDs, hydromorphone’s superior potency and flexibility in administration enable it to manage moderate-to-severe postoperative pain more effectively. Moreover, compared to sedative drugs (e.g., midazolam and propofol [14]), hydromorphone demonstrates greater effectiveness in simultaneously achieving rapid and sustained control of pain and agitation, both of which are crucial in postoperative settings.

Despite its efficacy, hydromorphone should be administered cautiously in children with OSA, as they may exhibit heightened sensitivity to opioids [16]. Hydromorphone is currently regarded as an alternative option for managing moderate to severe nociceptive pain, and precautions must be taken to prevent the inadvertent administration of hydromorphone when morphine is intended [19,36]. Strategies to minimize opioid consumption and avoid opioid misuse include:

(a) Before: artificial intelligence-based pre-anesthesia counseling [37,38], and preoperative educational animations for children.
(b) During: peri-operative multimodal analgesia: other promising candidates and adjuvants for controlling EA in children include nalbuphine [12], ketamine, and dexmedetomidine. Ketamine’s NMDA receptor antagonism and dexmedetomidine’s alpha-2 adrenergic receptor agonism provide both analgesic and sedative effects, making them effective in minimizing EA without significant side effects [39–41]. Local regional anesthesia and non-pharmacological techniques are also effective.
(c) After: immediate parental presence and comfort. Analgesic administration at home with self-report measures [42] and analgesic-associated telemedicine [43,44].

Several limitations were noted in the present study.

- This is a single-center study with a limited sample size.
- No quantitative assessment of pre-anesthesia anxiety (as baseline) in children and parents.
- The follow-up data of the present study are unable to provide information on long-term recovery including postoperative chronic pain and postoperative negative behaviors [11].
- The optimal dosage for hydromorphone administration during AT surgery remains undiscovered. Choosing hydromorphone dosage based on the severity of OSA may help develop a personalized regimen [22].
- The neuromuscular blockade had not been monitored; this may hinder the evaluation of outcomes by confusing EA with psychomotor agitation due to incomplete muscle relaxant reversal or re-curarization [45].

## 5 Conclusion

Hydromorphone at a dose of 30 μg/kg can reduce the incidence of EA within 30 min after extubation among children after AT surgery compared to fentanyl. Hydromorphone showed superior analgesic effect, and it did not increase the incidence of oversedation, respiratory depression, or other adverse effects.

## Supplementary Material

Supplementary Table
