# Supplementary material for: Hydromorphone reduced the incidence of emergence agitation after adenotonsillectomy in children with obstructive sleep apnea: A randomized, double-blind study
Source: Open Med (Wars). 2025 Feb 7;20(1):20241129. doi: 10.1515/med-2024-1129 (PMC11806236; doi:10.1515/med-2024-1129)
Supplement: Supplementary Table [file med-2024-1129-sm.pdf]

Supplementary material

Table S1: Comparison of PAED scores between two groups at different time points after transferring to PACU. Values are mean ± SD

Time points	Hydromorphone group (*n* = 93)	Fentanyl group (*n* = 93)	Mean difference (95%CI)	*t* value	*P* value
**Eyes open**	12.41 ± 5.22	13.32 ± 3.73	−0.91 (−2.23 ~ 0.4)	−1.37	0.171
**Postextubation**					
0 min	12.84 ± 5.18	13.26 ± 2.63	−0.42 (−1.61 ~ 0.77)	−0.7	0.487
10 min	9.51 ± 5.02	12.57 ± 4.57	−3.06 (−4.45 ~ −1.68)	−4.35	<**0.001**
20 min	7.95 ± 5.19	10.47 ± 5.88	−2.53 (−4.13 ~ −0.92)	−3.11	**0.002**
30 min	6.4 ± 5.56	8.34 ± 6.45	−1.95 (−3.69 ~ −0.2)	−2.2	**0.029**
**Exiting PACU**	5.15 ± 4.99	7.2 ± 6.51	−2.05 (−3.73 ~ −0.37)	−2.41	**0.017**
**Postoperative**					
2 h	3.25 ± 3.75	3.98 ± 4.66	−0.73 (−1.96 ~ 0.49)	−1.18	0.24
4 h	1.71 ± 2.67	2.73 ± 3.91	−1.02 (−1.99 ~ −0.05)	−2.08	**0.039**
6 h	1.01 ± 2.31	2.27 ± 3.54	−1.26 (−2.12 ~ −0.39)	−2.87	**0.005**

Abbreviations: PAED, pediatric anesthesia emergence delirium; PACU, post-anesthesia care unit; SD, standard deviation; CI, Confidence interval. [a]*P* value compares the hydromorphone group and fentanyl group. [b]*t*-test used to compare means of PAED scores.

Table S2: Comparison of FLACC scores between two groups at different time points after transferring to PACU. Values are mean ± SD

Time points	Hydromorphone group (*n* = 93)	Fentanyl group (*n* = 93)	Mean difference (95%CI)	*t* value	*P* value[a,b]
**Eyes open**	3.35 ± 3.55	3.85 ± 3.41	−0.49 (−1.50 ~ 0.51)	−0.97	0.334
**Postextubation**					
0 min	3.57 ± 3.62	2.86 ± 3.28	0.71 (−0.29 ~ 1.71)	1.40	0.163
10 min	1.32 ± 2.28	3.27 ± 3.81	−1.95 (−2.86 ~ −1.04)	−4.23	<**0.001**
20 min	1.02 ± 2.26	3.47 ± 3.69	−2.45 (−3.34 ~ −1.57)	−5.47	<**0.001**
30 min	1.02 ± 2.36	3.32 ± 3.49	−2.30 (−3.16 ~ −1.44)	−5.27	<**0.001**
**Exiting PACU**	0.83 ± 2.07	3.28 ± 3.46	−2.45 (−3.28 ~ −1.63)	−5.87	<**0.001**
**Postoperative**					
2 h	0.46 ± 1.22	1.01 ± 1.85	−0.55 (−1.00 ~ −0.09)	−2.39	**0.018**
4 h	0.33 ± 1.06	0.72 ± 1.60	−0.39 (−0.78 ~ 0.01)	−1.95	0.053
6 h	0.31 ± 1.17	0.52 ± 1.33	−0.20 (−0.57 ~ 0.16)	−1.11	0.268

Abbreviations: FLACC the face, legs, activity, crying, Consolability; PACU, post-anesthesia care unit; SD, standard deviation; CI, Confidence interval. [a]*P* value compares the hydromorphone group and fentanyl group. [b]*t*-test used to compare means of FLACC scores.

Table S3: Comparison of Ramsay scores between two groups at different time points after transferring to PACU. Values are mean ± SD

Time points	Hydromorphone group (*n* = 93)	Fentanyl group (*n* = 93)	Mean difference (95%CI)	*t* value	*P* value
**Eyes open**	2.01 ± 1.25	1.96 ± 1.29	0.05 (−0.31 ~ 0.42)	0.29	0.773
**Postextubation**					
0 min	2.11 ± 1.44	2.59 ± 1.77	−0.48 (−0.95 ~ −0.02)	−2.04	**0.042**
10 min	2.46 ± 1.29	2.29 ± 1.42	0.17 (−0.22 ~ 0.56)	0.87	0.388
20 min	2.46 ± 1.12	1.91 ± 1.13	0.55 (0.22 ~ 0.87)	3.33	**0.001**
30 min	2.19 ± 0.81	1.72 ± 0.67	0.47 (0.26 ~ 0.69)	4.35	<**0.0001**
**Exiting PACU**	2.02 ± 0.59	1.71 ± 0.65	0.31 (0.13 ~ 0.49)	3.42	**0.001**
**Postoperative**					
2 h	2.14 ± 0.72	1.98 ± 0.74	0.16 (−0.05 ~ 0.37)	1.51	0.132
4 h	2.13 ± 0.66	2.03 ± 0.54	0.1 (−0.08 ~ 0.27)	1.09	0.277
6 h	2.09 ± 0.50	2.09 ± 0.48	0 (−0.14 ~ 0.14)	0.00	1.000

Abbreviations: SD, standard deviation; PACU, post-anesthesia care unit. [a]*P* value compares the hydromorphone group and fentanyl group. [b]*t*-test used to compare means of Ramsay scores.
